# Multiclass EEG signal classification utilizing Rényi min-entropy-based feature selection from wavelet packet transformation

**DOI:** 10.1186/s40708-020-00108-y

**Published:** 2020-06-16

**Authors:** Md. Asadur Rahman, Farzana Khanam, Mohiuddin Ahmad, Mohammad Shorif Uddin

**Affiliations:** 1 Department of Biomedical Engineering, Military Institute of Science & Technology (MIST), Mirpur Cantonment, Dhaka, 1216 Bangladesh; 2 Department of Biomedical Engineering, Jashore University of Science and Technology (JUST), Jashore, 7408 Bangladesh; 3 Department of Electrical and Electronic Engineering, Khulna University of Engineering & Technology (KUET), Khulna, 9203 Bangladesh; 4 Department of Computer Science and Engineering, Jahangirnagar University, Dhaka, Bangladesh

**Keywords:** Electro-encephalogram (EEG), Brain–computer interface (BCI), Feature extraction, Wavelet packet transformation (WPT), Shannon entropy, Mutual information, Rényi min-entropy, Machine learning algorithms

## Abstract

This paper proposes a novel feature selection method utilizing Rényi min-entropy-based algorithm for achieving a highly efficient brain–computer interface (BCI). Usually, wavelet packet transformation (WPT) is extensively used for feature extraction from electro-encephalogram (EEG) signals. For the case of multiple-class problem, classification accuracy solely depends on the effective feature selection from the WPT features. In conventional approaches, Shannon entropy and mutual information methods are often used to select the features. In this work, we have shown that our proposed Rényi min-entropy-based approach outperforms the conventional methods for multiple EEG signal classification. The dataset of BCI competition-IV (contains 4-class motor imagery EEG signal) is used for this experiment. The data are preprocessed and separated as the classes and used for the feature extraction using WPT. Then, for feature selection Shannon entropy, mutual information, and Rényi min-entropy methods are applied. With the selected features, four-class motor imagery EEG signals are classified using several machine learning algorithms. The results suggest that the proposed method is better than the conventional approaches for multiple-class BCI.

## Introduction

Brain–computer interface (BCI) is a modern notion that enables the way of generating communication between computer and brain functionalities. It is not only expected, but also satisfactorily achieved technology that could be a nice solution to assist the physically challenged people to control devices utilizing their brain functionalities. The brain functionality can be assessed in two different ways: electrical activities (suggested modalities are electro-corticogram (ECoG), electro-encephalogram (EEG), and magneto-encephalogram (MEG)) and hemodynamics (functional near-infrared spectroscopy (fNIRS), functional magnetic resonance imaging (fMRI), etc.) [1, 2]. Based on the electrical activities, EEG is the most familiar, non-invasive, cheapest, and fastest modality for functional brain signal recording [3]. Among many brain stimuli, motor imagery (MI) movement is the highest choice for the researchers [2]. MI has a special benefit because it needs no additional setup like visual stimuli [3].

For providing the MI movement-based stimuli a candidate imagines the pattern of the real executive movements. During such MI movement, the EEG signals are recorded from the scalp of the participant that can be used to control the switches through a computer-based signal processing and this is broadly called BCI. There are different types of MI EEG-based BCI such as lifting hands and feet [4], simple–compound upper limb MI [5], uninterrupted hand movements [6] and finger movements [7], etc. Different stimuli show different classification performances. Especially, the upper limb shows more activeness than the lower limb MI movements. In addition, most of the EEG-MI research works [8–13] are related to two-class or three-class problem. Multiclass, i.e., more than 3-class classification for the EEG-MI signal is very challenging because of their non-discriminative features. To meet the challenge of 4-class EEG-MI, a very handful research works [14–16] were proposed where two hands MI, feet MI, and tongue movements were considered but their classification accuracy is not promising. This implies that they could not extract the required discriminative features in EEG signal through multiple stimuli. A very intelligent feature extraction and selection method could be an ultimate solution to meet the challenge of multiclass EEG signals.

There are a number of ways to extract features from EEG signals, such as autoregressive (AR) methods [17, 18], phase-space reconstruction approach [10], CSP-based methods [14, 16, 19], empirical mode decomposition [20–22], multivariate empirical mode decomposition-based methodologies [23, 24], channel correlation and feature optimization-based model [25], PCA-*t*-statistics-based feature extraction and selection method [26], wavelet transforms (WT) method [17, 27–30], etc. For a wide range of pattern recognition, wavelet packet transformation (WPT) provides excellent time–frequency features and therefore this approach is widely accepted feature extraction method for the EEG-MI movement classification. The WPT-based feature extraction has two important limitations: (i) structuring the features and (ii) selection of the bases. The features are structured by WPT coefficients those are considered to yield the significant pattern of the different classes EEG signal. Besides the feature structuring, proper base selection is the other step by which the structured features can show the highest discriminative characteristics among the classes. To overcome the limitations of WPT, Shannon entropy-based joint best basis method was proposed in [31], which is also questionable. This is because the proposed joint best basis method is effective in compression instead of classification [32]. To overcome the existing limitations in [31], a symmetric relative entropy-based local discriminant basis algorithm was proposed in [33]. It is reported in [34] that both methods rely on the signal’s energy level which exhibits hampering effect to achieve high classification for multiple classes. Eventually, it can be summarized that measuring the distance alone may not be wise consideration to judge the ability of the features for discriminating different classes [35]. Recent feature selection-based research works [23–25] reported their proposal on the BCI competition-IV dataset [36]. Correlation-based channel selection method with regularized common spatial pattern proposed in [25] did not cover the time–frequency characteristics of the EEG signal and on the other hand, the proposal given in [23, 24] utilized the time–frequency domain features but the feature selection method based on the Riemannian geometry could be further improved to achieve more accuracy in the classification of the MI events from the BCI competition-IV dataset. In this consequence, a more powerful feature selection algorithm is required to identify the meaningful content among the different features.

As it is explained in [37, 38] that the WPT computes more effectively than the WT and EMD in case of large size signal, we can choose WPT for feature extraction. In conventional approaches, Shannon entropy and mutual information method are often used to select the features found from the WPT coefficients. This work proposes to utilize the concept of Rényi min-entropy-based approach with a slight modification for the feature selection from the WPT coefficients. This method chooses features from a large feature set based on a special form of entropy compared to the Shannon entropy and mutual information method to attain the higher classification accuracy. Therefore, the main contributions of this work are:

- To extract WPT features from the EEG signals of four-class MI dataset.
- Selecting the features utilizing the proposed modified Rényi min-entropy-based approach.
- Comparing the classification performance of the proposed method with the conventional feature selection methods.
- To evaluate the classification performances of the proposed method using different classifiers.
- To compare the classification accuracy of the proposed method with the recent published proposals.

The dataset of BCI competition-IV (contains 4-class MI EEG signal) is used for this experiment. The data are preprocessed and separated in classes and used for the feature extraction using WPT. Then, for feature selection Shannon entropy, mutual information, and Rényi min-entropy methods are applied. With the selected features of 4-class motor imagery EEG signals are classified using several machine learning algorithms such as support vector machine (SVM), random forest, k-nearest neighbor (k-NN), multi-layer perceptron artificial neural network (MLP-ANN), logistic regression (LR), etc. Obtained results confirmed that the accuracy of the proposed method is higher than that of the Shannon entropy and mutual information. In addition, the proposed method also outperforms the recent state-of-the-art methods related to the applied dataset.

The rest of the paper is organized as follows: the materials and methods of this work are described with necessary steps in Sect. 2. In Sect. 3, the results are presented with required discussions; and research outcomes are concluded in Sect. 4.

## Materials and methods

### Data collections

**Fig. 1:**
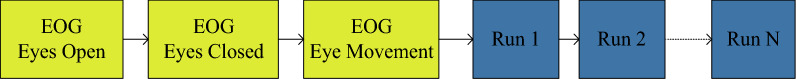
Timing scheme for each session

A multiclass valid and widely accepted datasets are taken in this study which is well known as BCI competition-IV. This dataset includes 9 healthy participants with no history of diseases and medications as well as they were verbally informed about the acquisition procedure and the possible outcomes of this research. Each participant performs different tasks based on a visual cue given on a screen. This cue-assisted data acquisition paradigm consisted of four motor imagery tasks: the imagination of movement of the left hand, right hand, both feet, and tongue. For each participant, two sessions (one for training and another for testing) on different days were recorded. Each session has 6 runs while one run contains 12 trials in each class. Therefore, one run produces (12 × 4=) 48 trials and each session contains total (48 × 6=) 288 trials. As one session is considered as training and another session is considered as testing, so we have 72 (= 288/4) trials of each class for training and the same number of trials for testing. Each data set is strongly influenced by EOG. Data were recorded using 25 channels (22 EEG channels and 3 EOG channels) with a sampling rate of 250 Hz. The data acquisition schedule is illustrated in Fig. 1. The original EEG data are downsampled to 100 Hz. The detailed explanation of the dataset is available in http://www.bbci.de/competition/iv/.

### Data preprocessing

**Fig. 2:**
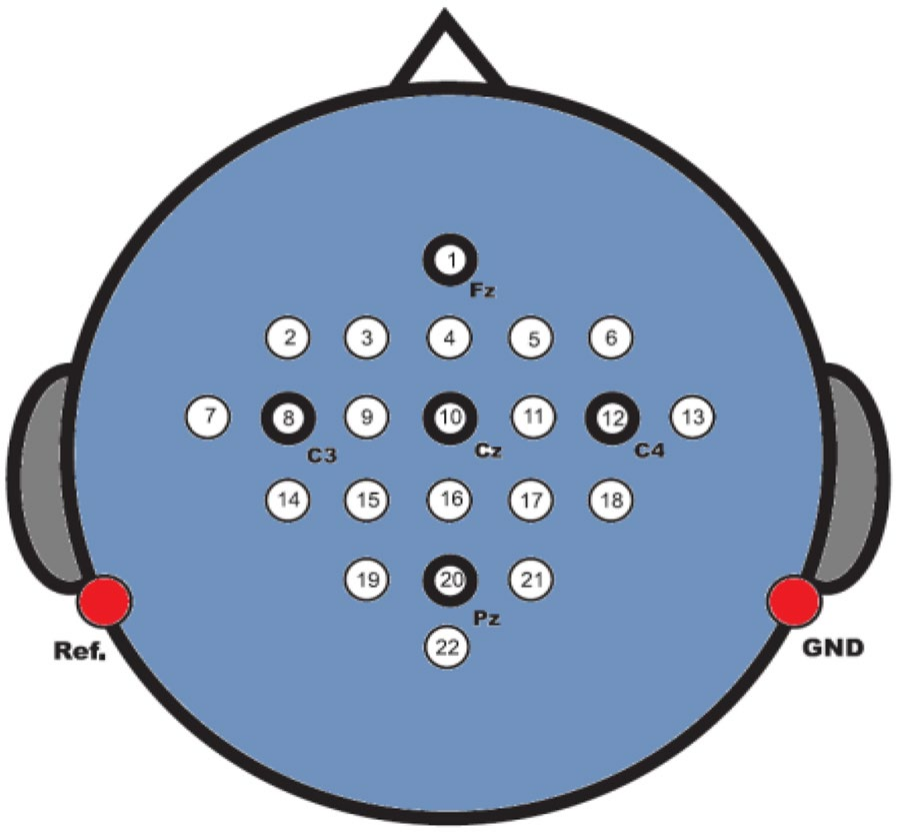
The channel number and their locations on the scalp of the used EEG modality

The sampling rate of the EEG signals of the used dataset is 250 Hz and the signals were previously filtered using a bandpass filter of frequency band within 0.5 and 100 Hz. The sensitivity of the amplifier was considered 100 mV. In addition, a 50-Hz notch filter was utilized to remove the line noise. Among 25 channels, 10 significant channels (channel no. 2, 3, 4, 5, 6, 8, 9, 10, 11, and 12) were considered as they represent the central and frontal region of the brain (the detailed positions of the channels are given in Fig. 2). Also, the effect of eye blink and EOG were removed utilizing the enhanced automatic wavelet independent component analysis (EAWICA) toolbox [39]. Finally, the EEG signals were separated according to the schedule of the tasks.

### Feature extraction using wavelet packet transformation

WPT differs from conventional wavelet transformation as it decomposes both approximate coefficients and detailed coefficients. We can compare WPT to a subspace tree. The original signal space represents the root node of the tree and it can be denoted as $\varPi_{0,0}$. The general form of this equation is $\varPi_{j,k}$, where notation *j* and *k* indicate the scale and the sub-band space. This original signal equation becomes $\varPi_{j,k} \to \varPi_{j + 1,2k}$ when it is decomposed into approximation space. In case of detailed space, the equation is $\varPi_{j,k} \to \varPi_{j + 1,2k + 1}$. The space decomposition idea is derived from the concept of dividing the orthogonal basis function of the original signal. Here, $\left\{ {\varphi_{j} (t - 2^{j} k)} \right\}_{k \in Z}$ denoting orthogonal basis function is transformed into two new orthogonal bases: (i) $\left\{ {\varphi_{j + 1} (t - 2^{j + 1} k)} \right\}_{k \in Z}$ of approximate space $\varPi_{j + 1,2k}$ and (ii) $\left\{ {\psi_{j + 1} (t - 2^{j + 1} k)} \right\}_{k \in Z}$ of detailed space $\varPi_{j + 1,2k + 1}$. Here $\varPi_{j,k} (t)$ and $\varPsi_{j,k}$ represent the scaling and wavelet functions, respectively. These functions are equated as [32]:

1 $$\varphi _{{j,k}} (t) = \frac{1}{{\sqrt {\left| {2^{j} } \right|} }}\varphi \left( {\frac{{t - 2^{j} k}}{{2^{j} }}} \right),$$

2 $$\psi _{{j,k}} (t) = \frac{1}{{\sqrt {\left| {2^{j} } \right|} }}\psi \left( {\frac{{t - 2^{j} k}}{{2^{j} }}} \right).$$

**Fig. 3:**
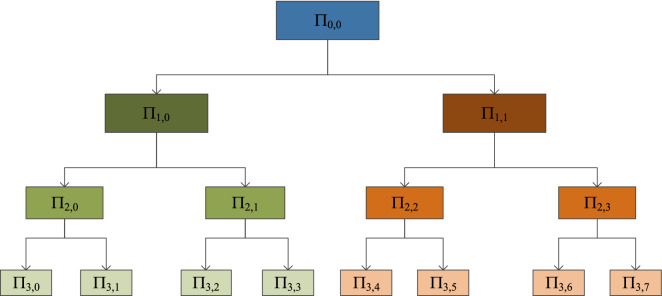
Wavelet packet decomposition mechanism with their 3-level coefficients

The scaling or compression degree of the original signal is calculated by the scaling parameter $2^{j}$. Moreover, $2^{j} k$ of these equations is named as a location parameter or translation parameter, which indicates the time location of the wavelet. The mentioned process can be repeated $J$ times, where $J$ must be less than $\log_{2} N$. $N$ of $\log_{2} N$ denotes the total number of samples in the original signal $\varPi_{j,k}$. The final outcome of WPT is $J \times N$ coefficients. Thus, the tree has ${N \mathord{\left/ {\vphantom {N {(2j)}}} \right. \kern-0pt} {(2j)}}$ number of coefficient blocks at any level of calculation *j*$\left[ {j = 1,2, \ldots ,J} \right]$. The iterative process adds more tree nodes to the WPT tree, where the nodes represent the subspace of different frequency localization characteristics. The corresponding decomposition procedure can be presented as Fig. 3 [4, 32, 40].

### Evolution of Rényi entropies

According to the usual definition, the Rényi entropies [41] are a type of functions that represent the uncertainty related to a random variable. The Rényi entropy is regarded as a non-negative real number (suppose, the order of the number is $\delta$; $\delta \ne 1$)), with $\delta \ne 1$, and is defined mathematically as:

3 $$H_{\delta } (X)\mathop = \limits^{\text{def}} \frac{1}{1 - \delta }\log \left( {\sum\limits_{i} {p(x_{i} )\delta } } \right).$$

It is clear to understand that in case of uniform value of $p( \cdot )$ the Rényi entropies are equal to $\log \left| X \right|$. Otherwise, the process will be as decreasing form in $\delta$. Particularly, we can define the Shannon and min-entropy as:

$$\delta \to 1 \, H_{1} (X) = - \sum\limits_{x} {p(x)\log p(x)} \;\;\;\;\left( {\text{Shannon entropy}} \right),$$

$$\delta \to \infty \, H_{\infty } (X) = - p(x)\log p(x)\;\;\;\;{ \text{min-entropy}}.$$

According to the Shannon approach, the conditional entropy of $X$ with respect to the given $Y$ provides the mean residual entropy of $X$ if the $Y$ value id given and mathematically we can represent it as:

4 $$H_{1} (X|Y)\mathop = \limits^{\text{def}} - \sum\limits_{xy} {p(x,y)\log p(x|y) = \,} H_{1} (X,Y) - H_{1} (Y).$$

In (4), $H_{1} (X,Y) = {\text{entropy}}\;{\text{of}}\;\;(X \cap Y).$ On the other hand, mutual information of Shannon approach regarding the previously proposed $X$ and $Y$ represents the correlation of information between $X$ and $Y$, and we can define it as:

5 $$I_{1} (X;Y)\mathop = \limits^{\text{def}} H_{1} (X) - H_{1} (X|Y) = H_{1} (X) + H_{1} (Y) - H_{1} (X,Y).$$

We can easily show that $I_{1} (X;Y) \ge 0$ along with $I_{1} (X;Y) = 0$ if we found that both $X$ and $Y$ are independent of each other, and hence $I_{1} (X;Y) = I_{1} (Y;X)$.

The fundamental proposal of Rényi was not supposed to define the conditional entropy as well as mutual information for the basic $\delta$. A nice proposal with conditional min-entropy associating the Rényi’s algorithm is given in [42] and the approach can be presented mathematically as:

6 $$H_{\infty } (X|Y)\mathop = \limits^{\text{def}} - \log \sum\limits_{y} {\mathop {\hbox{max} }\limits_{x} p\left( {(y|x)p(x)} \right)} .$$

It can be shown that this proposal is related to the Bayes risk. According to the method of Bayes risk, it is the error of guessing $X$ with the given value of $Y$ which can be defined mathematically as:

7 $$\beta (X|Y)\mathop = \limits^{\text{def}} 1 - \sum\limits_{y} {p(y)\mathop {\hbox{max} }\limits_{x} p(x|y)} .$$

Also, we define the mutual information as,

8 $$I_{\infty } (X;Y)\mathop { = }\limits^{\text{def}} H_{\infty } (X) - H_{\infty } (X|Y).$$

We can easily show that $I_{\infty } (X;Y) \ge 0$ along with $I_{\infty } (X;Y) = 0$ if we found that both $X$ and $Y$ are independent of each other (the reverse may not be true, that means $I_{\infty }$ is not symmetric). Therefore, the conditional mutual information in the case of this approach can be defined as, $I_{\infty } (X;Y|Z)\mathop = \limits^{\text{def}} H_{\infty } (X|Z) - H_{\infty } (X|Y,Z)$, which is also analogous to conditional mutual information of Shannon approach.

### Proposed feature selection algorithm

Suppose that the feature and class set are $F$ and $C$, respectively. Since the proposed algorithm is designed considering (i) forward feature selection and (ii) dependency maximization, it builds a continuously incremental sequence $\{ Q^{t} \}_{t > 0}$ of subsets of $F$. According to the necessity, at every phase, the subset $Q^{t + 1}$ is calculated by adding the next feature. It should be noted that the consideration of the “**order of importance**” is based upon the conditional min-entropy. An interactive test based on the stopping criteria is supposed to be performed for sequence construction on the achieved accuracy through the current subset for the multiclass problem. While we achieve the required accuracy level, the algorithm stops itself and provides the resulting subset $Q^{t}$. In this case, the accuracy level $1 - \varepsilon$ will possibly be found if the Bayes risk function gives as $\beta (C|F) < \varepsilon$.

We define the series $\{ Q^{t} \}_{t > 0}$ and $\{ f^{t} \}_{t > 0}$ inductively as given below:

9 $$Q^{0} \mathop = \limits^{def} \varphi ,$$

10 $$f^{t + 1} \mathop = \limits^{def} \arg \min_{{f \in F\backslash Q^{t} }} H_{\infty } (C|f,Q^{t} ),$$

11 $$Q^{t + 1} \mathop = \limits^{def} S^{t} \cup \{ f^{t + 1} \} .$$

The proposed algorithms in [43] and [44] are also similar although they used Shannon entropy. According to the mentioned algorithms in [43, 44], $f^{t + 1}$ is defined based on the maximization of the mutual information rather than the conditional entropy minimization. According to the proposal of [45] this is irrelevant because $I_{\infty } (C;f|Q^{t} ) = H_{\infty } (C|Q^{t} ) - H_{\infty } (C|f,Q^{t} )$. Therefore, maximizing $I_{\infty } (C;f|Q^{t} )$ with respect to $f$ is similar to minimizing $H_{\infty } (C|f,Q^{t} )$ with respect to $f$. This condition holds similar action in Shannon entropy. It can be proved that this proposal is locally optimal and the proof is given in Appendix.

## Results and discussion

**Fig. 4:**
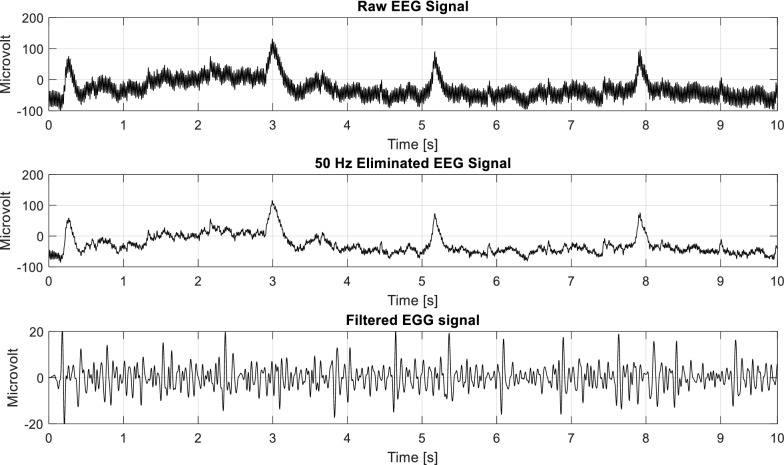
Original EEG signal and its stepwise filtering effect (according to the proposal) on the signal

**Fig. 5:**
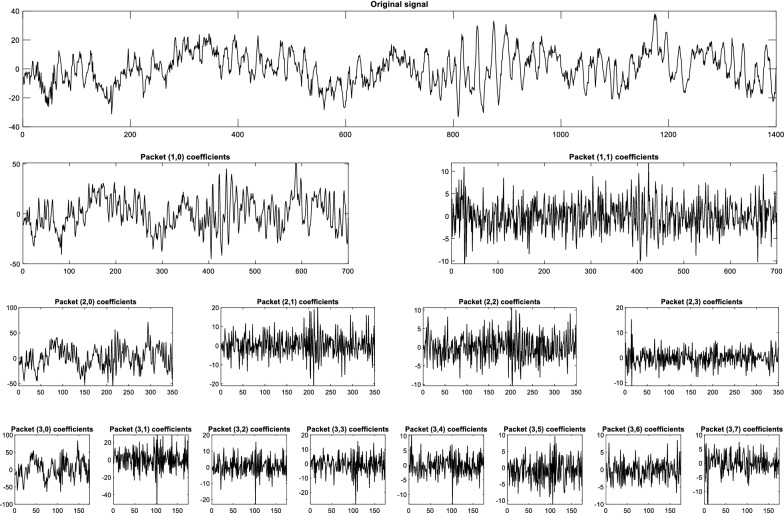
An example of the wavelet packet decomposition (up to level 3) on a randomly selected EEG signal of the utilized dataset

As the approach of the proposal, 4-class motor imagery EEG signals are collected from the BCI competition-IV. Although these signals were preprocessed, the signal was filtered with a 50-Hz notch filter and after that, the signals were again filtered to remove EOG effect by EWICA toolbox as described in preprocessing subsection of this article. The stepwise filtering effect from the original EEG signals is presented in Fig. 4. After that, the EEG signals are considered for dual-tree WPT. A randomly selected EEG signal is illustrated up to a 3-level WPT in Fig. 5. This figure illustrates the different frequency content-based signals of the used EEG signal. Since the EEG signals are separated according to the schedule of the previously mentioned 4-class MI tasks, the separated EEG signals were considered for feature extraction by WPT. With the help of WPT all EEG signals are decomposed up to 5 levels and extracted four different features (Energy, Variance, Standard Deviation, and Waveform Length) as proposed in [46]. Therefore, for level 5 decomposition, we can find 2^5^ = 32-type of features for each class. Eventually, every EEG signal will provide 32 × 4 = 128 features.

**Fig. 6:**
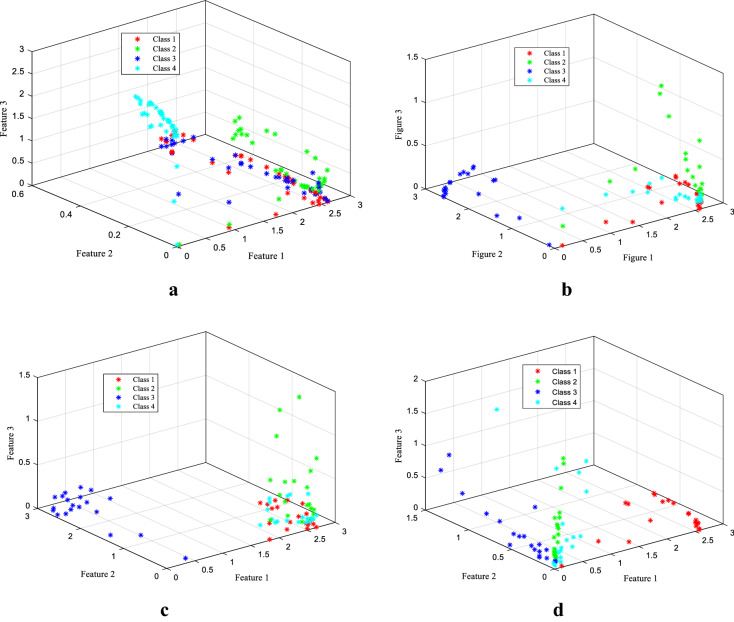
Representation of the differentiability of the different methods among the features in feature space. **a** All WPT features, **b** selected features by Shannon theory, **c** selected features by mutual information theory, and **d** selected features by Rényi’s min-entropy algorithm

According to the claim of this work, all these features are not necessary for the classifier. Therefore, we need to choose the correct features from them. In conventional procedures like Shannon theory and mutual information theory, we can reduce the feature dimensions. Having some limitations of the conventional procedure (explained in the previous subsections), the proposed work utilizes the method of Rényi’s min-entropy-based algorithm for feature selection. In the feature space, it is found that the proposed method extracts significant distinguished patterns from each other among the features. Figure 6 shows the features of the four classes in the feature space. From the figure, we get that the differentiability among the features in Rényi’s min-entropy is larger than the conventional methods (Shannon entropy and mutual information).

This representation of the features of different classes is the justification of the higher efficiency of the proposed method compared to the other conventional methods. Therefore, the training efficiency of the classifier would be better in the case of the proposed method and it can be predicted that the classification accuracy could also be higher than the other feature selection methods.

Since the training and testing data were previously separated in the used dataset, the features from training data were used to train the SVM classifier and the selected features of the testing EEG signals were utilized to test the classification accuracies. The average classification accuracies of SVM corresponding to the above-mentioned feature selection algorithms are given in Table 1. The results claim that the proposed Rényi’s min-entropy-based feature selection algorithm shows significantly higher classification accuracy than that of the conventional feature selection algorithms.

**Table 1:** The classification accuracy of the proposed algorithm compared to the others

Subject ID	Classification accuracy (%) of only WPT features	Classification accuracy (%) of selected WPT features by Shannon theory	Classification accuracy (%) of selected WPT features by mutual information theory	Classification accuracy (%) of selected WPT features by Rényi’s min-entropy algorithm
1	57	60	68	82
2	52	65	76	86
3	70	50	71	78
4	70	65	72	73
5	43	62	74	79
6	64	67	71	81
7	48	53	76	75
8	62	62	72	79
9	69	64	72	75
Average ± std	59.44 ± 10.02	60.88 ± 5.75	72.44 ± 2.55	78.66 ± 4.03

For thorough experimentation, we have taken the SVM classifier due to its wide acceptance. Furthermore, there are some commonly used classifiers such as random forest, k-NN, MLP-ANN, and LR and these classifiers could be used to justify their applicability and performances using our proposed feature selection algorithm. So, we did the experimentations. Every classifier is trained with the training dataset and tested 5 times with the testing dataset and taken the average classification accuracy. This result is given in Table 2. From this result, we get that random forest and MLP-ANN provides slightly higher classification accuracy than the SVM but k-NN and LR provides inferior results than the SVM. The results of the proposed work are also compared with the previous recent work that dealt with the four-class problem of BCI IV dataset and the result is given in Table 3. From the results, we have found that the proposed method shows higher accuracy than the previous work. There are some works [23, 24] those also dealt with BCI IV dataset, but their presentation of the classification accuracy is in the binary approach and six-tuple presentation (Left vs. Right, Left vs. Foot (LvF), Left vs. Tongue (LvT), Right vs. Foot (RvF), Right vs. Tongue (RvT), Foot vs. Tongue (FvT)) of the four-class classification problem. Therefore, is not possible to compare this result with the proposed work directly.

**Table 2:** The classification accuracy of the different classifiers after adopting the proposed algorithm

Subject ID	SVM	Random forest	k-NN	MLP-ANN	LR
1	82	82	74	80	74
2	86	86	72	82	72
3	78	84	68	84	78
4	73	78	70	74	64
5	79	79	69	78	68
6	81	86	70	85	72
7	75	75	65	85	74
8	79	80	72	79	69
9	75	75	68	74	60
Average ± std	78.66 ± 4.03	80.55 ± 4.24	69.77 ± 2.68	80.11 ± 4.28	70.11 ± 5.53

**Table 3:** The average classification accuracy of the proposed work with the recent state-of-the-art

Proposed algorithm + random forest	Proposed algorithm + MLP-ANN	Jin et al. [25]
80.55	80.11	78%

## Conclusions

The dual-tree wavelet decomposition of the EEG signals is a nice way to extract features for the EEG-based motor imagery-related task classification. However, more levels of decomposition create a number of features for multiple classes that become a burden for a classifier and hence the resulting classification accuracy reduces. Therefore, an intelligent feature selection algorithm is necessary to reduce the feature number, and consequently, it would be necessary to increase the discriminating power of the features. This research work has proposed and utilized the Rényi’s min-entropy algorithm along with a slight modification to select the WPT features for getting the higher classification accuracies. A four-class MI EEG signal of BCI competition-IV dataset is used to justify the proposed work, and from the results we found that the proposed method outperforms 18% and 6% increment in classification accuracy (in average) than the Shannon entropy and mutual information methods, respectively, in case of SVM classifier. On the other hand, applying the random forest and MLP-ANN the classification accuracy could be increased up to 8% with respect to mutual information methods. Since the performance is too convincing, this intelligent feature selection algorithm will hopefully open a new pathway to implement multiple-class BCI in practice.

## Data Availability

This work utilizes the open data source BCI competition-IV which is available in [36].

## Appendix: Proof of the proposed method as an optimal feature selection algorithm

The previously stated set $Q^{t + 1}$ reduces to the Bayes risk related to the classification among the probabilities those are of the form $Q^{t} \cup \{ f\}$, known mathematically as:

12 $$\forall f \in F\beta (C|Q^{t + 1} ) \le \beta (C|Q^{t} \cup \{ f\} ).$$

### *Proof:*

Suppose that, $\bar{\theta }$, $\theta$, $\theta^{\prime}$ represent common value tuples and values of $Q^{t}$, $f$, and $f^{t + 1}$, respectively,

13 $$\sum\limits_{{\bar{\theta },\theta }} {\mathop {\hbox{max} }\limits_{c} } \left( {p(\bar{\theta },\theta |c)p(c)} \right) \le \sum\limits_{{\bar{\theta },\theta^{\prime}}} {\mathop {\hbox{max} }\limits_{c} } \left( {p(\bar{\theta },\theta^{\prime}|c)p(c)} \right).$$

Using the Bayes theorem (12), we get

14 $$\sum\limits_{{\bar{\theta },\theta }} {p(\bar{\theta },\theta )\mathop {\hbox{max} }\limits_{c} } p(c|\bar{\theta }) \le \sum\limits_{{\bar{\theta },\theta }} {p(\bar{\theta },\theta^{\prime})\mathop {\hbox{max} }\limits_{c} } p(\bar{\theta },\theta^{\prime}|c).$$

Then, from the definition of Bayes risk, we get the following relation (15):

15 $$\beta (C|Q^{t} \cup \{ f^{t + 1} \} ) \le \beta (C|Q^{t} \cup \{ f\} ).$$
